# The Great Masquerader: Vasospastic Angina Mimicking Left Main Coronary Artery Disease

**DOI:** 10.3390/jcm15051952

**Published:** 2026-03-04

**Authors:** Maja Wojtylak, Katarzyna Frączek, Aleksander Zeliaś, Tomasz Tokarek

**Affiliations:** 1 Center for Innovative Medical Education, Jagiellonian University Medical College, Medyczna 7, 30-688 Kraków, Poland; maja.wojtylak@student.uj.edu.pl (M.W.); katarzyna.fraczek.2000@gmail.com (K.F.); 2 Students’ Scientific Group of Center for Innovative Medical Education, Jagiellonian University Medical College, 30-688 Kraków, Poland; 3 Faculty of Medicine and Health Science, University of Applied Science in Nowy Sącz, 33-300 Nowy Sącz, Poland; a.zelias@intercard.net.pl; 4 2nd Clinical Department of Cardiology, Faculty of Medicine and Health Science, University of Applied Science in Nowy Sącz, 33-300 Nowy Sącz, Poland; 5 Center for Invasive Cardiology, Electrotherapy and Angiology, Kilinskiego 68, 33-300 Nowy Sącz, Poland

**Keywords:** angina, INOCA, coronary microvascular dysfunction, vasospastic angina, coronary flow reserve, index of microcirculatory resistance

## Abstract

A significant proportion of patients undergoing invasive coronary angiography for angina have no obstructive coronary artery disease (CAD). In such patients, coronary microvascular dysfunction (CMD) and vasospastic angina (VSA) represent key pathophysiological mechanisms. We report a case of a 58-year-old male with exertional chest pain and exercise ECG changes typical of left main or multivessel CAD. Coronary computed tomography angiography (CCTA) showed borderline stenosis of the distal left main coronary artery. Coronary angiography revealed no critical stenosis. A comprehensive functional assessment demonstrated reduced coronary flow reserve (CFR = 2.0) and an elevated index of microcirculatory resistance (IMR = 25), consistent with CMD. An intracoronary acetylcholine provocation test induced severe focal vasospasm of the mid-left anterior descending artery (LAD) with ST-segment elevation and anginal pain, promptly relieved by nitroglycerin, confirming VSA. This case highlights the diagnostic and clinical importance of invasive functional testing in patients with angina and non-obstructive coronary arteries (ANOCA/INOCA). The coexistence of CMD and VSA (two distinct but overlapping pathophysiological endotypes) is increasingly recognized as a marker of adverse prognosis. Functional coronary assessment should be considered in all patients with angina and non-obstructive coronary arteries, as identifying mixed endotypes enables precise, mechanism-guided therapy.

## 1. Introduction

In registries, up to 70% of patients with persistent angina symptoms and non-obstructive coronary arteries on angiography (ANOCA) have evidence of myocardial ischemia (INOCA). The underlying mechanisms are heterogeneous and include coronary microvascular dysfunction (CMD), manifesting as microvascular angina, and epicardial coronary vasospasm (vasospastic angina, VSA) [1,2,3]. Both entities may coexist and are associated with recurrent symptoms, impaired quality of life, repeated hospitalizations, and worse prognosis compared with isolated endotypes [2,3]. Historically, the absence of obstructive coronary artery disease (CAD) on angiography often led to diagnostic uncertainty, symptom underestimation, and inappropriate reassurance. However, contemporary data clearly demonstrate that ANOCA/INOCA is not a benign condition [2,3,4,5]. Patients frequently experience persistent angina, psychological distress, repeated investigations, and elevated risk of adverse cardiovascular outcomes. This has led to a paradigm shift from purely anatomical assessment toward functional and pathophysiological characterization of coronary circulation. Invasive coronary function testing, including assessment of epicardial stenosis severity, microvascular resistance, coronary flow reserve, and vasoreactivity, have emerged as the reference standard for defining coronary vasomotor disorders. Recent trials and registries have shown that mechanism-guided therapy based on functional testing significantly improves symptoms, quality of life, and clinical outcomes compared with empirical treatment [1,2,3,4,5,6,7].

Recent data derived from cohorts of patients with persistent angina following PCI further underscore the pivotal role of coronary function testing (CFT) in elucidating the underlying pathophysiological substrate and facilitating targeted, mechanism-based therapy. In these populations, a substantial proportion of symptoms are attributable not to residual epicardial obstruction but to endothelial dysfunction, vasospastic angina, or coronary microvascular dysfunction, highlighting the limitations of anatomy-driven management strategies alone [8] Additionally, current studies have demonstrated that heterogeneity within the group of ANOCA/INOCA patients necessitates a multimodal, physiology-guided approach within the diagnostics, including multivessel CFT [9,10]. Furthermore, the rapid evolution of advanced computational techniques, particularly the integration of artificial intelligence into cardiovascular imaging, offers additional opportunities to refine non-invasive risk stratification and optimize downstream testing strategies in patients without obstructive CAD. Nevertheless, despite these technological advances, invasive functional assessment remains indispensable for the definitive evaluation of coronary microvascular dysfunction and vasospastic angina [11].

Herein, we present a patient with symptoms strongly mimicking obstructive left main CAD, supported by high-risk exercise ECG changes and borderline findings on coronary f(CCTA). Conventional coronary angiography revealed no critical stenosis, but invasive functional and provocation testing allowed precise diagnosis of concurrent CMD and VSA.

## 2. Case Presentation

**Figure 1:**
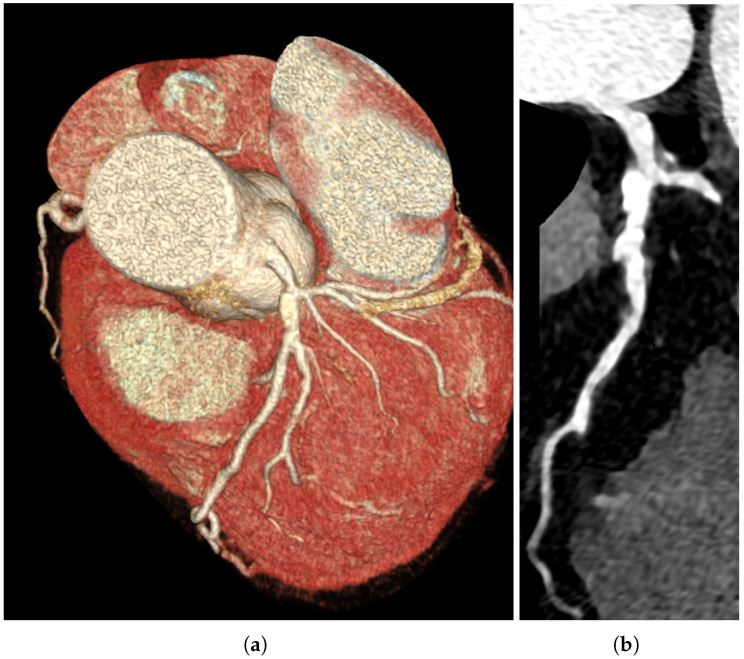
(**a**,**b**) Computed tomography angiography with borderline left main coronary artery stenosis with estimated minimal lumen area of 7 mm^2^.

A 58-year-old man presented with a recent onset of retrosternal chest pain, usually triggered by exercise. His past medical history was significant for arterial hypertension, dyslipidemia, gastro-esophageal reflux disease, and obesity. Two years earlier, coronary angiography revealed 30% stenosis at the distal part of the left main coronary artery (LMCA) with no significant atherosclerosis changes in other vessels. A CCTA showed borderline stenosis (30–40%) in the distal LMCA with an estimated minimal lumen area of 7 mm^2^ (Figure 1).

**Figure 2:**
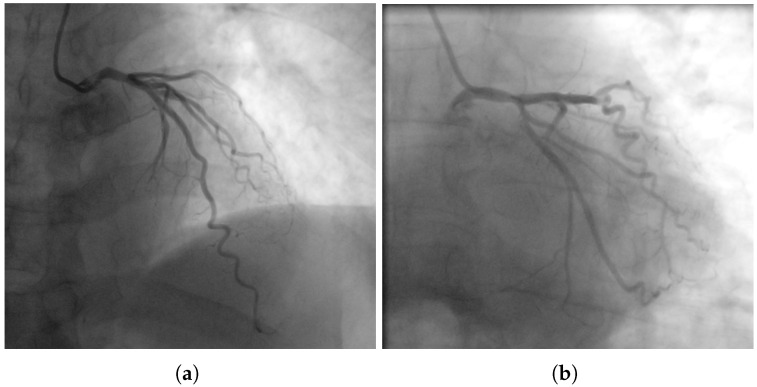

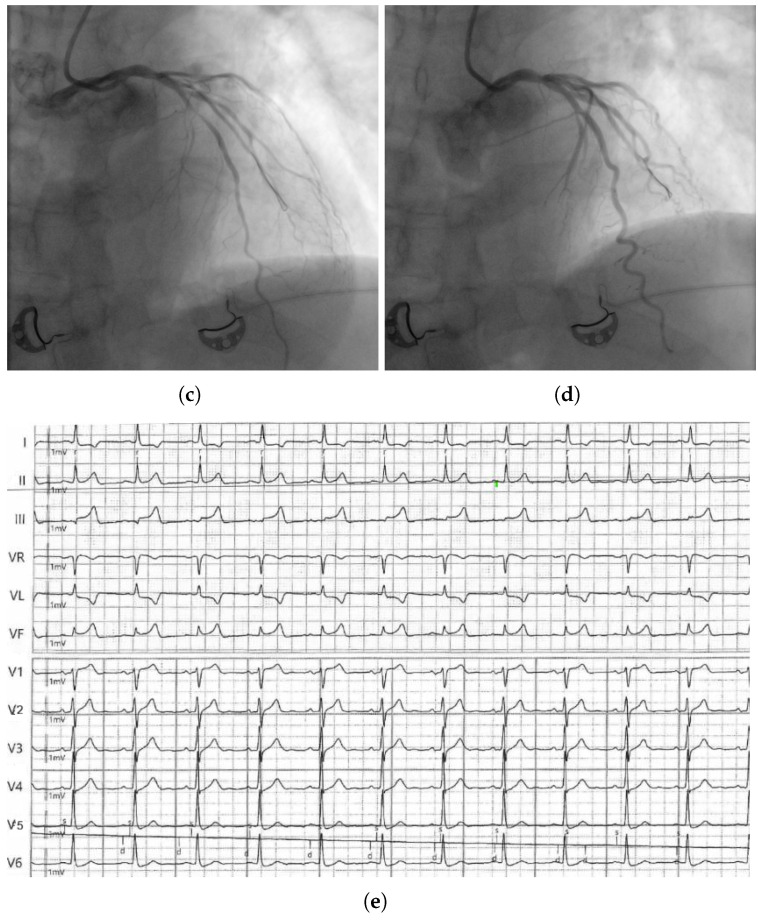
(**a**,**b**) Coronary angiography with no hemodynamically significant stenoses. (**c**,**d**) Severe focal vasospasm in the middle segment of LAD during the intracoronary acetylcholine provocation test. (**e**) ECG during the acetylcholine provocation test with ST-segment elevation in leads II, III, and aVF, with reciprocal depression in leads I, aVL, and V5–V6. Letters **s** and **d**—systole and diastole in the cardiac cycle determined by automatic ECG.

During the treadmill exercise test, crushing retrosternal pain and diaphoresis occurred after a short effort; the ECG showed ST-segment elevation in lead aVR and V1 with reciprocal ST-segment depression in other leads, strongly suggesting obstructive CAD. The patient was admitted urgently; coronary angiography showed no hemodynamically significant stenoses (Figure 2a,b). Given the discordance between anatomical and clinical findings, a comprehensive functional assessment was performed.

Continuous intravenous adenosine infusion at 140 μg/kg/min was used to achieve maximal hyperemia. Pressure and flow measurements were obtained in the distal segment of the left anterior descending artery (LAD). The resting full-cycle ratio (RFR) and fractional flow reserve (FFR) values were 0.92 and 0.85, respectively, indicating the absence of flow-limiting epicardial stenosis. Subsequently, microvascular indices were assessed: the coronary flow reserve (CFR) was 2.0 and the index of microcirculatory resistance (IMR) was 25, fulfilling the diagnostic criteria for coronary microvascular dysfunction (CFR < 2.0 and/or IMR ≥ 25), as defined in a contemporary expert consensus document [4].

Furthermore, an intracoronary acetylcholine (ACh) provocation test was performed. Incremental ACh doses induced a severe focal vasospasm in the middle segment of LAD, resulting in approximately 95% lumen narrowing (Figure 2c,d), accompanied by ST-segment elevation in leads II, III, and aVF, with reciprocal depression in leads I, aVL, and V5–V6 (Figure 2e). The patient developed typical anginal pain, which resolved rapidly after administration of intracoronary nitroglycerin, confirming a nitrate-responsive epicardial spasm consistent with VSA [5]. The patient was initiated on targeted pharmacotherapy addressing both components of coronary vasomotor dysfunction, including isosorbide mononitrate, molsidomine, and non-dihydropyridine calcium channel blocker (diltiazem). No symptoms were reported on routine six-month follow-up in the outpatient department.

## 3. Discussion

This case illustrated the complexity and clinical importance of diagnosing coronary vasomotor disorders in patients with ANOCA. The striking discordance between dramatic exercise ECG changes suggestive of left main disease and a lack of critical stenosis in coronary angiography underscores the limitations of anatomical imaging alone. CMD and VSA represent distinct but overlapping pathophysiological entities. CMD is characterized by impaired microvascular dilatation and/or increased microvascular resistance, leading to ischemia despite angiographically normal arteries. VSA results from transient, intense epicardial coronary constriction, causing dynamic obstruction and myocardial ischemia. The coexistence of both mechanisms, as demonstrated in this patient, is increasingly recognized in the contemporary literature as a marker of adverse prognosis, associated with recurrent angina, impaired quality of life, and higher risk of major adverse cardiovascular events [3,4,5,7]. Several contemporary studies have demonstrated that up to 50% of patients with ANOCA have CMD, VSA, or both when systematically evaluated with invasive functional testing [1,2,3,4,5]. The CorMicA trial showed that stratified therapy guided by invasive coronary function testing significantly improved angina severity and quality of life compared with standard care [3]. These findings have influenced international guidelines, and the 2024 ESC Guidelines on Chronic Coronary Syndromes now recommend invasive functional testing in selected patients with persistent angina despite non-obstructive coronary arteries [3,7]. These data underline a paradigm shift from purely anatomical to mechanism-guided diagnosis and treatment.

The present case exemplifies a diagnostically complex scenario in which borderline anatomical findings—specifically a 30–40% LMCA stenosis on CCTA—coexisted with exercise-induced ST-segment depressions suggestive of multivessel ischemia. Despite this apparently high-risk non-invasive profile, invasive coronary angiography did not confirm hemodynamically significant epicardial obstruction. Instead, comprehensive coronary function testing demonstrated vasospastic angina with concomitant microcirculatory dysfunction, underscoring a clear anatomical-functional discordance. This observation highlights the limitations of relying solely on stenosis severity, particularly in intermediate LMCA lesions, when determining the need for revascularization.

The coexistence of CMD and VSA represents a challenging clinical phenotype, thus tailored pharmacological strategy is required. While calcium channel blockers and nitrates are first-line therapy for vasospastic angina, microvascular dysfunction often requires additional strategies, including beta-blockers, ACE inhibitors, ranolazine, trimetazidine, or lifestyle-based interventions. Risk factor modification is crucial, as hypertension, dyslipidemia, smoking, obesity, and psychosocial stress strongly influence microvascular and vasomotor function [1,2,3,4,5,6,7].

This case also highlights the psychological and clinical burden carried by patients with unexplained angina. Before functional testing, many patients experienced repeated hospitalizations, unnecessary stent implantation or diagnostic uncertainty. Establishing a clear mechanistic diagnosis not only guides therapy but also improves patient understanding, adherence, and quality of life. From a prognostic perspective, ANOCA is not benign. Long-term studies demonstrate that patients with CMD or VSA have higher rates of myocardial infarction, heart failure, stroke, and cardiovascular death compared with asymptomatic individuals. The presence of mixed endotypes further increases this risk, supporting the need for early recognition and targeted management [2,3,4,5].

Comparable patterns have been described in contemporary cohorts of patients with persistent angina, including those evaluated after PCI, in whom endothelial dysfunction, epicardial spasm, and coronary microvascular dysfunction are highly prevalent despite the absence of flow-limiting disease. These data reinforce the principle that ischemic symptoms and objective evidence of ischemia may reflect functional coronary disorders rather than residual or progressive epicardial obstruction, and therefore should not automatically prompt further mechanical intervention [8].

Recent data also demonstrate that multivessel coronary function testing increases diagnostic yield in patients with INOCA, as coronary vasomotor abnormalities may be heterogeneous across different vascular territories [9]. Furthermore, systematic reviews suggest that the reported prevalence of coronary microvascular dysfunction varies considerably depending on the diagnostic modality applied, supporting a multimodality and physiology-guided approach [10].

Advanced computational approaches, including AI-driven plaque quantification and CT-FFR, may help bridge this anatomical-functional gap by providing lesion-specific physiological assessment directly from CCTA datasets. Such techniques have demonstrated growing utility even in complex scenarios, including prior stenting, and may reduce unnecessary invasive procedures triggered by anatomically intermediate but functionally insignificant lesions. Importantly, in patients with ANOCA, these tools may assist in distinguishing between true flow-limiting epicardial disease and functional coronary disorders, thereby optimizing downstream testing strategies [11].

Nevertheless, it should be emphasized that while CT-FFR enhances the non-invasive assessment of epicardial lesion significance, it does not evaluate coronary vasomotor function or microcirculatory integrity. Therefore, in patients with persistent symptoms and non-obstructive coronary arteries, invasive coronary function testing remains indispensable for identifying vasospastic angina and coronary microvascular dysfunction. A stepwise approach integrating anatomical imaging, AI-assisted physiological modeling, and targeted invasive functional testing may represent the most rational contemporary strategy in complex ANOCA presentations [11].

Collectively, these considerations support a stepwise, physiology-guided strategy integrating high-resolution anatomical imaging, AI-assisted functional modeling, and targeted invasive vasomotor assessment. Such an approach enhances diagnostic precision, prevents unnecessary PCI in the setting of borderline anatomical stenosis, and facilitates mechanism-directed therapy in complex ANOCA presentations [11].

This case reinforces several key clinical lessons:

1. Severe ischemic symptoms and high-risk ECG changes may occur in the absence of obstructive CAD.
2. Functional coronary testing is essential when symptoms and angiography are discordant.
3. Mixed CMD and VSA is a high-risk phenotype requiring individualized therapy.
4. Mechanism-guided treatment can lead to excellent symptom control and improved outcomes.

Future research should focus on refining diagnostic algorithms, identifying non-invasive surrogates of coronary dysfunction, and developing targeted therapies for specific endotypes. Nevertheless, invasive functional testing currently remains the reference standard for precise phenotyping of coronary vasomotor disorders.

## 4. Conclusions

This case demonstrates that a normal or non-obstructive coronary angiogram does not exclude clinically significant coronary disease. Comprehensive functional evaluation can reveal microvascular dysfunction and/or epicardial vasospasm that perfectly mimic obstructive multivessel or left main coronary disease. Identifying overlapping vasomotor endotypes provides diagnostic clarity, improves patient understanding, and enables mechanism-based therapy. Tailored treatment addressing both epicardial and microvascular components can markedly reduce symptoms and may improve long-term prognosis. Routine consideration of coronary functional testing in selected patients with persistent angina and non-obstructive coronary arteries represents a critical step toward personalized, evidence-based cardiovascular care.

## Data Availability

The original contributions presented in this manuscript are included in the references section. Further inquiries can be directed to the corresponding author.
